# Associations between sun sensitive pigmentary genes and serum prostate specific antigen levels

**DOI:** 10.1371/journal.pone.0193893

**Published:** 2018-03-08

**Authors:** Visalini Nair-Shalliker, Sam Egger, Agata Chrzanowska, Rebecca Mason, Louise Waite, David Le Couteur, Markus J. Seibel, David J. Handelsman, Robert Cumming, David P. Smith, Bruce K. Armstrong

**Affiliations:** 1 Cancer Research Division, Cancer Council New South Wales, Sydney, Australia; 2 Sydney School of Public Health, The University of Sydney, Sydney, Australia; 3 Department of Clinical Medicine, Macquarie University, Sydney, Australia; 4 Sydney Medical School, The University of Sydney, Sydney, Australia; 5 Centre for Education and Research on Ageing, Concord Hospital and The University of Sydney, Sydney, New South Wales, Australia; 6 ANZAC Research Institute, The University of Sydney, Sydney, New South Wales, Australia; 7 Menzies Health Institute Queensland, Griffith University, Southport, Queensland, Australia; 8 School of Population Health, University of Western Australia, Perth, Western Australia, Australia; Carolina Urologic Research Center, UNITED STATES

## Abstract

**Background:**

Melanoma and prostate cancer may share risk factors. This study examined the association between serum PSA levels, which is a risk factor for prostate cancer, and variants in some melanoma-associated pigmentary genes.

**Methods:**

We studied participants, all aged 70+ years, in the Concord Health and Ageing in Men Project who had no history of prostatitis or received treatment for prostate disease (n = 1033). We genotyped variants in MC1R (rs1805007, rs1805008), ASIP (rs4911414, rs1015362), SLC45A2 (rs28777, rs16891982), IRF4 (rs12203592), TYRP1 (rs1408799), TYR (rs1126809, rs1042602), SLC24A2 (rs12896399), and OCA2 (rs7495174). Generalised linear dominant models with Poisson distribution, log link functions and robust variance estimators estimated adjusted percentage differences (%PSA) in mean serum PSA levels (ng/mL) between variant and wildtype (0%PSA = reference) genotypes, adjusting for age, body mass index, serum 25OHD levels and birth regions (Australia or New Zealand (ANZ), Europe or elsewhere).

**Results:**

Serum PSA levels were strongly associated with advancing age and birth regions: mean PSA levels were lower in Europe-born (-29.7%) and elsewhere-born (-11.7%) men than ANZ-born men (reference). Lower %PSA was observed in men with variants in SLC45A2: rs28777 (-19.6;95%CI: -33.5, -2.7), rs16891982 (-17.3;95%CI:-30.4,-1.7) than in wildtype men (reference). There were significant interactions between birth regions and PSA levels in men with variants in MC1R (rs1805007; p-interaction = 0.0001) and ASIP (rs4911414; p-interaction = 0.007). For these genes %PSA was greater in ANZ-born men and lower in Europe- and elsewhere-born men with the variant than it was in wildtype men. In a post hoc analysis, serum testosterone levels were increased in men with MC1R rs1805007 and serum dihydrotestosterone in men with ASIP rs1015362.

**Conclusion:**

Men with SNPs in SLC45A2, who have less sun sensitive skin, have lower PSA levels. Men with SNPs in MC1R and ASIP, who have more sun sensitive skin, and were born in ANZ, have higher PSA levels. Androgens may modify these apparent associations of pigmentary genes and sun exposure with PSA levels.

**Impact:**

PSA levels and possibly prostate cancer risk may vary with sun sensitivity and sun exposure, the effects of which might be modified by androgen levels.

## Introduction

Prostate cancer (PC) is the most commonly diagnosed cancer in men in developed countries, with Australia estimated to have the highest age standardised incidence rates in the world (151/100,000 in 2012) [1, 2]. Established predictors of PC include advancing age, African ancestry, and family history of PC [3].

There is growing evidence that cutaneous melanoma (CM) and some of its predictors are also predictors of PC risk. Data from 15 cancer registries in different countries showed a relative risk (RR) for PC of 1.27 (95%CI: 1.20–1.33) in men diagnosed with cutaneous melanoma; similar findings have been reported in Australian and Norwegian men (under review) [4, 5]. Solar ultraviolet radiation (UVR), an established predictor for melanoma, may also be a predictor of PC: studies from regions of high ambient UV levels have reported positive associations between UVR and PC risk, while inverse associations were reported in regions of low ambient UV [6–9]. Additionally, other predictors of melanoma risk, such as pigmentary characteristics and variants in pigmentary genes, have been shown to be associated with PC risk, albeit inconsistently. Red-haired participants in the Alpha-Tocopherol, Beta-Carotene Cancer Prevention Study were half as likely to develop PC as other men [10]. Results from the Prostate Testing for Cancer and Treatment study (ProtecT) study found that men with a high proportion of variants in pigmentary genes that increase sun sensitivity had greater PC risk than men with no such variants [11]. There is no clear mechanism for the associations between these factors and PC risk.

Serum prostate specific antigen (PSA) is uniquely produced by the prostate gland. Elevated serum PSA levels suggest increased prostate cell activity, possibly as a result of cancer, but are more commonly due to non-cancerous causes such as ethnicity, older age, benign prostatic hyperplasia, inflammation, infection, recent sexual activity and recent physical activity [3, 12, 13]. Considering serum PSA levels as an indicator of prostate cancer risk [13], this study examined the associations between serum PSA levels and some of the variants in pigmentary genes previously shown to be associated with melanoma risk. We also examined these associations separately by region of birth because of substantial observed differences in frequency of pigmentary gene variants and mean blood PSA levels by region of birth.

## Materials and methods

All analyses were based on baseline information collected by The Concord Health and Ageing in Men Project (CHAMP), which is a population-based longitudinal study of older men. Details of this study are described elsewhere [12]. CHAMP was approved by the Sydney South West Area Health Service Human Research Ethics Committee–Concord Repatriation General Hospital Zone, and the present analysis of CHAMP data was approved by the University of Sydney Human Research Ethics Committee (#10428) and the Cancer Council New South Wales Human Research Ethics Committee (#218). A written informed consent was obtained from each participant before blood collection and clinic examination at baseline [12].

### Study population

Eligible CHAMP participants were men 70 years of age and older and living in the Burwood, Strathfield and Canada Bay local government areas of Sydney at the time of recruitment. Men were identified through the NSW electoral roll and invited by mail to participate between January 2005 and June 2007. Men were ineligible to participate if residing in nursing homes or found to have died or moved from the area before the initial contact attempt. Of potentially eligible men, 47% participated [14].

All participants were mailed a questionnaire before their first research clinic visit; it included, among others, demographic questions, prostate health questions, and questions on type, duration and frequency of physical activity. Completed questionnaires were collected during the first clinic visit.

For the current analysis, all men who reported having ever had any inflammation or infection of the prostate, or having received treatment for any prostate disease, were excluded.

### Serum analysis

Fasting blood samples were collected from participants on the morning of their clinic visit. Total PSA analyses were carried out by the Central Sydney Area Health Service laboratory at Concord Repatriation General Hospital on the day of collection, using electrochemiluminescence immunoassay on the Modular Analytics E170 (Elecsys module) immunoassay analyzer (Roche Diagnostics GmbH, D-68298 Mannheim). The intra-assay coefficient of variation was 2.73% at 4 ng/L and 2.71% at 37.6 ng/L. The standard reference range for the PSA assay was 0.0 to 4.0 ng/mL.

Sera for vitamin D assays were stored frozen at -80°C until the end of sample collection, after which all analyses for vitamin D were performed simultaneously. Serum 25OH levels were measured by manual RIA using single batch reagents (DiaSorin Inc., Stillwater, MN). The assay for 25(OH)D has a sensitivity of <1.5 ng/mL with an intra-assay precision of 7.6% and an inter-assay precision of 9.0%. The assays measure metabolites of both vitamin D_2_ and vitamin D_3,_ and were done in duplicate.

Sera were previously assayed for testosterone and dihydrotestosterone and details of this assay are described elsewhere [15] (see reference to post-hoc analysis below).

### DNA extraction and genotyping

DNA was extracted from 1 to 1.5 ml of buffy coat, collected at baseline. Genomic DNA extraction was performed using Qiagen genomic DNA extraction kit (Qiagen, Hilden, Germany) in accordance with the manufacturer’s instructions.

Custom genotyping assays were designed for SNPs that were selected based on studies showing strong associations between variants in pigmentation genes and CM risk [11, 16]. The following 11 SNPs from the pigmentation genes were analysed: Agouti signalling protein (ASIP: rs1015362, rs4911414), melanocortin-1-receptor (MC1R: rs1805007, rs1805008), melanoma antigen AIM1 (SLC45A2: rs28777, rs16891982), interferon regulatory factor 4 (IRF4: rs12203592), tyrosinase related protein 1 (TYRP1: rs1408799), tyrosinase (TYR: rs1126809, rs1042602), solute carrier family 24 member 2 (SLC24A2:rs12896399), and oculocutaneous albinism type 2 (OCA2: rs7495174). Ten percent of samples were added as duplicates so that concordance between genotype calls could be assessed. The genotyping assays were performed at the Australian Genome Research Facility (Brisbane) genotyping facilities. Samples containing 10ng/μL genomic DNA were genotyped using iPLEX Gold chemistry on a MALDI-TOF Compact Mass Spectrometer (Sequenom Inc, San Diego), by the Australian Genome Research Facility. The products were processed and analysed in a Compact Mass Spectrometer by MASSArray Workstation (Version 3.3) software (Sequenom Inc, San Diego). The overall genotyping pass rate was 97.3%

### Data analysis

A dominant inheritance model was used to assess the association of PSA with each SNP. The percentage difference (%PSA) between mean PSA in men with one or two copies of each SNP and mean PSA in men who were homozygous for the common variant of the SNP (wildtype, the reference group with its %PSA set to 0%) was estimated. Serum PSA levels were log-transformed. Serum 25OHD levels were categorised as sufficient at 50 nmol/L and above and insufficient at below 50 nmol/L. Body mass index (BMI) was grouped as healthy weight below 25 kg/m^2^ and overweight or obese at 25 kg/m^2^ and above according to the World Health Organisation criteria for being overweight or obese [3]. Region of birth was categorised as born in Australia or New Zealand (ANZ), Europe or elsewhere (outside ANZ and Europe).

Responses to the question on avoidance of direct sunlight (Never, Usually, Always) were used to compare sun avoidance behaviour between all participants, by region of birth, using those born in ANZ as the reference category.

### Statistical analysis

Generalised linear models with Poisson distribution, log link functions and robust variance estimators were used to estimate %PSA adjusted for age (per 5 years increase in age), BMI, birth regions, and serum 25OHD levels. Nominal p-values are shown for each statistical test with no adjustments made for multiple comparisons. The interactions between each SNP and region of birth were evaluated by including appropriate interaction terms in the regression models, and considered statistically significant if p for interaction was <0.05. Participants with missing data on age, BMI, PSA, serum 25OHD or region of birth data were excluded from analyses requiring those data. An increase in PSA levels was denoted by a plus (+) sign and a decrease by a minus (-) sign.

### Post-hoc analysis

Because associations of SNPs with PSA levels that we report here might be mediated by circulating male sex hormones, we undertook a post-hoc analysis of baseline measures of mean serum testosterone and dihydrotestosterone levels in men with rs1805007 (MC1R), rs4911414 (ASIP), rs28777 (SLC45A2) and rs16891982 (SLC45A2) compared to levels in men with the corresponding wildtypes, adjusting for age, 25OHD, BMI and birth regions. We used the same analytical methods as described above for PSA levels.

All analyses were performed using Stata 13.

## Results

Of the 1705 CHAMP participants, 672 were excluded from this analysis because of self-reported prostatitis (n = 154), having received treatment for prostate disease (n = 383), and missing age, BMI, PSA, serum 25OHD, region of birth or SNP-typing results (n = 135). There was a complete data set for 1033 men (S1 Fig).

The median age was 76 years (range 70 to 96). Serum PSA levels increased with age (p = 0.0001; Table 1): mean PSA was 3.2 ng/mL (median = 1.9 ng/mL) and increased 2.7% (95%CI:1.2, 4.2) on average for every year increase in age. Fifty percent of men were born in Australia or New Zealand (ANZ) (n = 512), 40% were born in Europe (n = 415) and the remaining 10% elsewhere (n = 106). Men born elsewhere comprised those born in South and West Asia (n = 39), East Asia (n = 40) and other parts of the world outside ANZ and Europe (n = 27). Serum PSA levels were also associated with region of birth (p<0.0001), with Europe- and elsewhere-born men having 29.7% (95%CI: -40.8, -16.4) and 11.7% (95%CI: -36.2,22.1) lower PSA levels, respectively, than ANZ-born men. There was no substantial association between PSA levels and serum 25OHD (p = 0.19) or BMI (p = 0.76).

**Table 1:** Cross-sectional analysis of the % differences in mean serum PSA level according participant characteristics.

Characteristic	N (%)	Mean PSA (ng/mL)	Adjusted % difference in mean PSA ^1^(95% CI)	p-value
All participants	1033 (100%)	3.2		
**Age (years)**				
Median (range)	76 (70 to 96)		+2.7 (1.2, +4.2) ^2^	<0.001
**Region of birth**				
ANZ	512 (50%)	3.7	ref.	<0.001
Europe	415 (40%)	2.5	-29.7 (-40.8, -16.4)	
Elsewhere^3^	106 (10%)	3.2	-11.7 (-36.2, +22.1)	
**25OHD (nmol/L)**				
Median (range)	54 (3 to 219)		+0.3 (-0.2, 0.8) ^2^	0.199
**All 3 regions of birth**				
<50	431 (42%)	2.9	ref.	0.190
≥50	602 (58%)	3.3	+11.9 (-5.4, 32.2)	
***ANZ***				
*<50*	178 (17%)	3.5	ref.	0.385
*≥50*	334 (32%)	3.8	+11.7 (-12.9, +43.2)	
***Europe***				
*<50*	198 (19%)	2.5	ref.	0.956
*≥50*	217 (21%)	2.5	-0.6 (-20.0, +23.4)	
**Elsewhere**^3^				
*<50*	55 (5%)	2.4	ref.	0.101
*≥50*	51 (8%)	3.9	+49.1 (-7.5, +140.3)	
**BMI (kg/m**^**2**^**)**				
Median (range)	28 (17 to 60)		+0.4 (-1.5, +2.4) ^2^	0.662
**All 3 regions of birth**				
<25	207 (20%)	3.3	ref.	0.763
≥25	826 (80%)	3.1	+2.9 (-14.5, +23.9)	
***ANZ***				
*<25*	117 (11%)	3.7	ref.	0.861
*≥25*	395 (38%)	3.7	+2.1 (-18.9, +28.5)	
***Europe***				
*<25*	48 (5%)	2.1	ref.	0.107
*≥25*	367 (36%)	2.5	+23.4 (-4.4, +59.2)	
**Elsewhere**^3^				
*<25*	42 (4%)	3.7	ref.	0.893
*≥25*	64 (6%)	2.8	-3.8 (-45.7, +70.2)	

^1^ Adjusted for age, plasma 25OHD level, BMI and region of birth.

^2^% difference per unit increase characteristic, where an increase in PSA levels was denoted by a plus (+) sign and a decrease by a minus (-) sign.

^3^ Men not born in Australia, New Zealand or Europe.

The prevalence of each SNP was examined in the three regions of birth (Table 2). Compared to ANZ-born men, Europe-born men were less likely to have variants in MC1R (rs1805007), ASIP (rs4911414), IRF4 (rs12203592), SLC24A4 (rs12896399) and TYR (rs1126809), and more likely to have them in ASIP (rs1015362), SLC45A2 (rs28777 and rs16891982), TYR (rs1042602) and OCA2 (rs7495174). Men born elsewhere were less likely than ANZ-born men to have all the listed pigmentary SNPS, except for the variants in SLC45A2 (rs28777 and rs16891982), TYRP1 (rs1408799) and OCA2 (rs7495174).

**Table 2:** Odds ratios (OR) and 95% confidence intervals (95%CI) for single nucleotide polymorphism in the pigmentary gene by region of birth.

Function	SNP	Australia		Europe		Elsewhere^1^		p-value^3^
		% WM, MM^2^	OR (ref)	% WM, MM^2^	OR (95%CI)	% WM, MM^2^	OR (95%CI)	
SLC45A2	rs28777	6.1	**1.00**	19.4	3.67 (2.37, 5.69)	69.5	34.88 (20.08, 60.58)	<0.001
SLC45A2	rs16891982	7.5	**1.00**	22.3	3.56 (2.38, 5.34)	74.3	35.81 (20.69, 61.97)	<0.001
MC1R	rs1805007	14.7	**1.00**	9.1	0.58 (0.38, 0.88)	2.8	0.17 (0.05, 0.55)	0.001
ASIP	rs4911414	56.8	**1.00**	49.3	0.74 (0.57, 0.96)	30.2	0.33 (0.21, 0.52)	<0.001
ASIP	rs1015362	42.4	**1.00**	48.8	1.29 (1.00, 1.68)	34.9	0.73 (0.47, 1.13)	0.020
IRF4	rs12203592	39.5	**1.00**	20.6	0.40 (0.30, 0.54)	8.6	0.14 (0.07, 0.29)	<0.001
SLC24A4	rs12896399	70.9	**1.00**	61.8	0.67 (0.50, 0.88)	62.3	0.68 (0.44, 1.05)	0.010
TYR	rs1042602	57.8	**1.00**	68.8	1.61 (1.22, 2.11)	26.4	0.26 (0.16, 0.42)	<0.001
TYR	rs1126809	54.1	**1.00**	42.7	0.63 (0.49, 0.82)	14.3	0.14 (0.08, 0.25)	<0.001
TYRP1	rs1408799	54.2	**1.00**	55.0	1.03 (0.79, 1.34)	83.0	4.13 (2.41, 7.06)	<0.001
OCA2	rs7495174	12.5	**1.00**	30.5	3.06 (2.18, 4.28)	53.8	8.11 (5.10, 12.88)	<0.001

^1^ For men not born in Australia, New Zealand or Europe.

^2^ MM = two copies of the variant and WM = one copy of the variant

^3^p-value for test of equal ORs across regions of birth

Only SNPs in SLC45A2 were significantly associated with PSA levels in all men (Table 3): lower %PSAs were observed in men with rs28777 (-19.6; 95%CI: -33.5, -2.7; p-value = 0.025) and rs16891982 (-17.3; 95%CI:-30.4,-1.7; p-value = 0.031). The associations of PSA levels with one SNP in MC1R (rs1805007, associated with red hair) and one SNP in ASIP (rs4911414, associated with high sun sensitivity) varied significantly among the three birth regions (p_interaction_ = 0.001 and 0.007 respectively; Table 3). The %PSA with rs1805007 (MC1R) was +23.7 (95%CI: -6.2, +63.1) for ANZ-born men, -45.6 (95%CI:-59.8,-26.3) for Europe-born men and -21.4 (-59.0,+50.4) for elsewhere-born men. The %PSA in men with rs4911414 (ASIP) was +33.7 (95%CI: +9.8,+62.9) for ANZ-born men, -9.7 (95%CI:-27.2,+12.0) for Europe-born men and -40.4 (95%CI:-66.4,+5.8) for elsewhere-born men.

**Table 3:** Adjusted percent difference in mean serum prostate specific antigen level in men with one or two variant pigmentary gene alleles compared to men with no variant alleles in the three birth regions.

Gene	SNP	Alleles (variant vs. ref)	% difference in mean PSA (95% CI)^2^				*p-*interaction^3^
			All birth regions^1^	ANZ	Europe	Elsewhere	
SLC45A2	rs28777	CA-CC vs. AA	**-19.6 (-33.5, -2.7)**	**-21.0 (-40.5, +4.9)**	-23.8 (-40.2, -2.9)	-7.6 (-47.1, +61.4)	0.808
SLC45A2	rs16891982	GC-CC vs. GG	**-17.3 (-30.4, -1.7)**	**-22.5 (-39.7, -0.3)**	-18.8 (-35.7, +2.5)	+3.7 (-42.3, +86.4)	0.738
MCIR	rs1805007	CT-TT vs. CC	+3.4 (-17.9, +30.2)	+23.7 (-6.2, +63.1)	-45.6 (-59.8, -26.3)	-21.4 (-59.0, +50.4)	**0.001**
ASIP	rs4911414	GT-TT vs. GG	+9.7 (-5.3, +27.2)	+33.7 (9.8, +62.9)	-9.7 (-27.2, +12.0)	-40.4 (-66.4, +5.8)	**0.007**
ASIP	rs1015362	GA-AA vs. GG	+6.7 (-9.3, +25.6)	+19.3 (-4.3, +48.7)	-6.3 (-24.4, +16.2)	-20.7 (-56.4, +44.1)	0.23
IRF4	rs12203592	CT-TT vs. CC	+11.8 (-6.2, +33.2)	+12.7 (-10.2, +41.6)	-2.3 (-24.4, +26.1)	+91.6 (0.3, +265.9)	0.182
OCA2	rs7495174	AG-GG vs. AA	+0.3 (-15.5, +19.0)	-17.0 (-36.7, +9.0)	+14.0 (-10.0, +44.4)	+12.9 (-34.3, +93.8)	0.225
TYR	rs1042602	CA-AA vs. CC	+7.8 (-7.6, +25.8)	+11.0 (-11.3, +38.9)	+13.3 (-8.6, +40.4)	-25.7 (-55.5, +24.0)	0.382
TYR	rs1126809	GA-AA vs. GG	-3.5 (-18.4, +14.2)	+1.7 (-19.9, + 29.2)	-15.2 (-31.2, +4.5)	-3.2 (-51.7, +94.0)	0.446
SLC24A4	rs12896399	GT-TT vs. GG	+5.9 (-9.8, +24.2)	+12.7 (-10.6, +42.2)	+0.7 (-20.8, +28.0)	-12.1 (-48.9, +51.0)	0.771
TYRP1	rs1408799	TC-TT vs. CC	-8.0 (-21.5, +8.0)	-2.4 (-22.2, +22.5)	-13.6 (-30.1, +6.9)	-26.2 (-58.9, +32.7)	0.61

^1^ Adjusted for age, serum 25OHD level, BMI and region of birth

^2^ Adjusted for age, serum 25OHD level and BMI

^3^*p*-interaction is p-value for the interaction test of whether adjusted % difference in mean PSA differs according to region of birth.

An analysis of sun exposure (always avoid versus never avoid) in these groups of men showed that Europe-born (OR = 2.25;95%CI:1.35–3.67) and elsewhere-born men (OR = 4.12;95%CI:2.06–8.22) were more likely to avoid direct sunlight than ANZ-born men. However, a sub-group analysis of SNPs in the pigmentary genes showed no material association with sun avoidance behaviour (data not shown).

In the post hoc analysis, only rs1805007 (MC1R) and rs1015362 (ASIP) were associated with male hormone levels after adjusting for age, serum 25OHD, BMI and birth regions (Table 4). The %testosterone was +8.7 (95%CI:1.0,16.8) in men with rs1805007 (MCIR). There was also a significant interaction of this association with birth region (p_interaction_ = 0.042). The %testosterone was highest in rs1805007-positive Europe-born men +14.7 (95%CI: -1.9, +29.1), a little less in ANZ-born men +6.9 (95%CI -2.6, +17.3) and least in elsewhere-born men -21.1 (95%CI -43.0,+9.2). The %dihydrotestosterone was +9.6% (95% CI +0.5, +19.5) in men with rs1015362 (ASIP) and there was no significant interaction with birth region (p_interaction_ = 0.44).

**Table 4:** Adjusted^1^ percent difference in mean testosterone and dihydrotestosterone levels in men with one or two variant pigmentary gene alleles compared to men with no variant alleles.

Gene	SNP	Alleles (variant vs. ref.)	% difference in mean testosterone (95% CI)				*p-*interaction^3^
			All regions^1^	ANZ^2^	Europe^2^	Elsewhere^2^	
MCIR	rs1805007	CT-TT vs. CC	**8.7 (1.0, 16.8)**	6.9 (-2.6, 17.3)	**14.7 (1.9, 29.1)**	-21.1 (-43.0, 9.2)	**0.042**
ASIP	rs4911414	GT-TT vs. GG	0.7 (-4.1, 5.7)	0.6 (-6.3, 8.1)	2.4 (-4.7, 10.1)	-6.5 (-20.2, 9.5)	0.577
ASIP	rs1015362	GA-AA vs. GG	3.4 (-1.5, 8.5)	3.7 (-3.4, 11.4)	4.2 (-3.2, 12.1)	-4.6 (-17.6, 10.5)	0.788
SLC45A2	rs28777	CA-CC vs. AA	1.5 (-4.9, 8.3)	8.9 (-2.4, 21.4)	-0.6 (-9.1, 8.6)	7.6 (-8.9, 27.2)	0.361
SLC45A2	rs16891982	GC-CC vs. GG	0.8 (-5.4, 7.4)	4.6 (-5.8, 16.0)	0.2 (-8.3, 9.4)	9.4 (-9.3, 32.0)	0.705
			**% difference in mean DHT (95% CI)**				**p-interaction**^4^
			**All regions**^1^	**ANZ**^2^	**Europe**^2^	**Elsewhere**^2^	
MCIR	rs1805007	CT-TT vs. CC	9.8 (-2.3, 23.5)	9.0 (-7.2, 27.9)	14.7 (-3.1, 35.8)	-23.1 (-55.0, 31.3)	0.243
ASIP	rs4911414	GT-TT vs. GG	3.2 (-4.7, 11.6)	10.3 (-2.9, 25.3)	-3.0 (-12.6, 7.6)	-6.1 (-22.0, 13.1)	0.235
ASIP	rs1015362	GA-AA vs. GG	**9.6 (0.5, 19.5)**	**15.6 (-0.1, 33.8)**	2.8 (-7.5, 14.2)	6.5 (-10.9, 27.2)	0.442
SLC45A2	rs28777	CA-CC vs. AA	-0.3 (-9.0, 9.2)	8.8 (-7.4, 27.9)	-3.2 (-14.9, 10.2)	4.6 (-15.9, 30.1)	0.474
SLC45A2	rs16891982	GC-CC vs. GG	-0.8 (-9.3, 8.4)	1.8 (-12.7, 18.7)	-0.4 (-12.3, 13.1)	5.1 (-16.8, 32.8)	0.845

^1^Adjusted for age, serum 25OHD level, and BMI region of birth

^2^Adjusted for age, serum 25OHD level, and BMI

^3^*p*-interaction is p-value for the interaction test of whether adjusted % difference in mean testosterone differs according to region of birth.

^4^*p*-interaction is p-value for the interaction test of whether adjusted % difference in mean dihydrotestosterone differs according to region of birth.

## Discussion

Cutaneous melanoma is a potential predictor of prostate cancer risk and there is growing evidence that variants in the melanoma associated pigmentary genes may mediate this effect [7, 9, 11, 17]. We observed a relationship between some SNPs in melanoma-associated pigmentary genes and serum PSA level, which is biomarker of risk for prostate cancer, which we believe to be novel. Two SNPs in SLC45A2 gene, rs2877 and rs16891982, which are more common with darker pigmentary phenotypes, were associated with lower PSA levels in all men. There was, in addition, quite strong evidence for interactions between variants in MC1R (rs1805007) and ASIP (rs4911414) and birth regions, in determining PSA levels, with ANZ-born men with these genotypes having higher PSA levels and Europe-born and elsewhere-born having lower PSA levels.

Elevated serum PSA levels suggest that prostate cancer might be present. Although mean PSA levels in our cohort of men, whose ages ranged between 70 and 96 years, was 3.2 ng/mL, almost 15% of participants had PSA levels above 5.5 ng/mL (data not shown). While PSA testing for early detection of prostate cancer is not recommended for men 70 and older in Australia, levels this high (and possibly even as low as 4.0 ng/mL) would be likely to trigger investigations for prostate cancer. The associations with birth regions, as proxy for ethnicity, were reasonably consistent with studies that reported associations between PSA levels and ethnicity, where levels in Asians were generally lower than Caucasians and this may have a biological basis [18–21]. Mean PSA levels in our study were highest in ANZ-born men. Although ANZ men would have been of predominantly European ancestry, based on 2001 Australian and New Zealand census, their levels were almost 30% higher than Europe-born men. These results suggest that ethnicity and, perhaps, sun exposure, which is higher in ANZ- than Europe- and elsewhere-born NSW residents may influence PSA levels [21, 22]. Following adjustments for possible confounding by age and birth regions, and additionally for body mass index and serum vitamin D levels, for potential confounding [11, 20], the significant associations were still evident.

We know of no previous studies that have specifically examined associations between pigmentary genotypes and PSA levels. We note though that no pigmentary genotypes were reported as associated with PSA levels with genome-wide significance (p-values all <5.8E-06) in a recent GWAS of PSA levels [23]. We were unable to find complete results from this study in which to search for results relating to the genotypes we studied. What of relevance do we know about the sun sensitivity genotypes that we found to be associated with PSA levels? SLC45A2 is a membrane transporter gene involved in melanin production. The rs16891982 variant of this gene is associated with dark hair and dark skin in Caucasians, and thus probably photoprotective [24]. Evidence of its link with CM risk is inconsistent but, on balance, favours a protective association [25–29]. The MC1R gene, located on the surface of melanocytes, plays an important role in producing eumelanin, which is responsible for synthesising brown or black pigments, and pheomelanin, which is responsible for synthesising yellow pigment [30]. Loss of MC1R function, e.g. presence of an inactivating MC1R gene variant, which rs1805007 is, favours pheomelanin over eumelanin production, and increases propensity to sunburn, and is associated with increased CM risk [31–33]. ASIP is an MC1R antagonist and therefore also associated with sun sensitivity. The ASIP SNPs, rs4911414 and rs1015362, are associated with increased sun sensitivity and with increased skin cancer risk [32, 34, 35]. Thus the functional effects of the SNPs we found to be associated with PSA levels are consistent with higher PSA levels in sun sensitive people, and vice versa.

The interactions between birth regions and some of the sun sensitivity SNPs may also suggest a role for sun exposure. In vitro studies have reported that high UVR exposure of melanocytes causes long-term repression of MC1R mRNA while moderate or low UVR exposure causes only temporary repression. In our cohort, ANZ-born men were least likely to avoid direct sunlight compared to the other groups of men, which suggests, consistently with other data, they have higher sun exposure than the Europe-born and elsewhere-born groups. Thus, sun exposure may offer a possible mechanism for the interaction observed between rs1805007 and region of birth in determining PSA level [36, 37].

Androgen regulation plays a role in both prostate carcinogenesis and melanomagenesis [38–40]. Testosterone is irreversibly converted to dihydrotestosterone (DHT), which are the two major androgens in humans. DHT has a higher potency than testosterone for stimulating proliferation and secretory function in the prostate [41, 42]. There may also be relationships between UVR exposure, androgen regulation, and MC1R gene regulation. A study in Xiphophorus fish, which is prone to UVR-caused melanoma, showed up-regulation of androgen levels after UV-B exposure [43]. Another study in cultured normal human melanocytes showed low testosterone levels down-regulated MC1R mRNA levels and decreased melanin production [37]. Thus, androgen levels may mediate the effect of UV-exposure on MC1R gene function. In our post-hoc analysis, testosterone and DHT levels were both elevated in men with MC1R and ASIP, but men with variants in SLC45A2, found in men with low propensity to burn, showed no association with either of the androgen levels (Table 4). This suggests potential cross-talk between epidermal and endocrine function, where the association between melanoma associated pigmentary genes and PSA levels may be mediated by epidermal androgen activity. Increased epidermal androgen activity may increase circulating androgen levels and systemically regulate organs that are responsive to androgens, such as the prostate [38].

It is strength of this study that most predictors of PSA levels were adjusted for in the analysis. The selection of the study population excluded men with a history of prostate disease. Also, predictors of PSA levels such as age, and region of birth as proxy for ethnicity, and potential predictors such as BMI and serum 25OHD levels, were adjusted for in the regression model. We chose not to adjust for multiple comparisons, and instead to evaluate the results in the context of prior evidence, biological plausibility, the number of tests performed, and the strengths of the observed associations, as recommended by a number of experts in this field [44–46]. We do acknowledge that whilst p-values are nominal for individual tests, the Type I error rate is likely to be inflated for the family of tests.

## Conclusion

Men with SNPs in SLC45A2, who have less sun sensitive skin, have lower PSA levels, whereas men with SNPs in MC1R and ASIP, which are associated with sun sensitive skin, and were born in in ANZ, which is associated with higher lifetime UVR exposure, have higher PSA levels. It is possible that androgens modify these apparent associations of pigmentary genes and sun exposure with PSA levels.

## Supporting information

S1 Fig Flow diagram showing final derivation of participants from the original CHAMP cohort. (TIF)
