# Supplementary figures and images for: Associations between sun sensitive pigmentary genes and serum prostate specific antigen levels
Source: PLoS One. 2018 Mar 8;13(3):e0193893. doi: 10.1371/journal.pone.0193893 (PMC5843239; doi:10.1371/journal.pone.0193893)

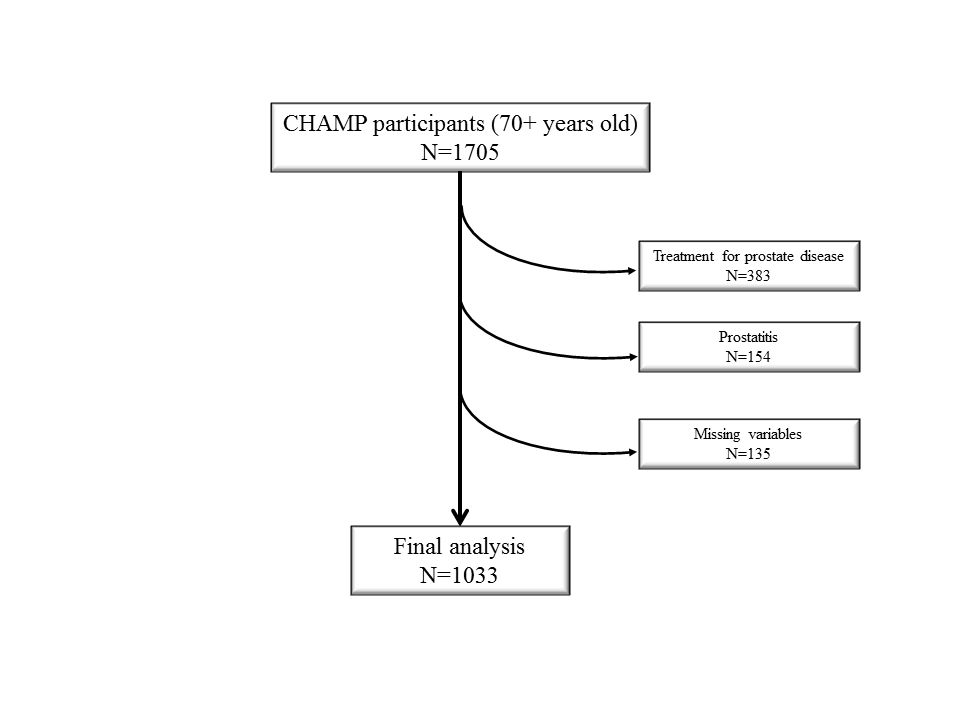

Supplement: S1 Fig — (TIF) [file pone.0193893.s001.tif]
